# Repair of articular cartilage and subchondral defects in rabbit knee joints with a polyvinyl alcohol/nano-hydroxyapatite/polyamide 66 biological composite material

**DOI:** 10.1186/s13018-017-0666-0

**Published:** 2017-11-15

**Authors:** Tao Guo, Xiaobin Tian, Bo Li, Tianfu Yang, Yubao Li

**Affiliations:** 1 Department of Orthopedics, Guizhou Province People’s Hospital, Guiyang, Guizhou province 550002 China; 2 Department of Orthopedics, West China Hospital, Sichuan University, Chengdu, Sichuan province 610041 China; 3 Nanometer Analytical and Testing Center, Sichuan University, Chengdu, Sichuan province 610041 China

**Keywords:** Biological composite material, Articular cartilage, Subchondral bone, Repair

## Abstract

**Background:**

This study sought to prepare a new PVA/n-HA/PA66 composite to investigate the repair of articular cartilage and subchondral defects in rabbit knee joints.

**Methods:**

A 5 × 5 × 5 mm-sized defect was created in the patellofemoral joints of 72 healthy adult New Zealand rabbits. The rabbits were then randomly divided into three groups (*n* = 24): PVA/n-HA+PA66 group, polyvinyl alcohol (PVA) group, and control (untreated) group. Cylindrical PVA/n-HA+PA66, 5 × 5 mm, comprised an upper PVA layer and a lower n-HA+PA66 layer. Macroscopic and histological evaluations were performed at 4, 8, 12, and 24 weeks, postoperatively. Type II collagen was measured by immunohistochemical staining. The implant/cartilage and bone interfaces were observed by scanning electron microscopy.

**Results:**

At 24 weeks postoperatively, the lower PVA/n-HA+PA66 layer became surrounded by cartilage, with no obvious degeneration. In the PVA group, an enlarged space was observed between the implant and the host tissue that had undergone degeneration. In the control group, the articular cartilage had become calcified. In the PVA/n-HA+PA66 group, positive type II collagen staining was observed between the composite and the surrounding cartilage and on the implant surface. In the PVA group, positive staining was slightly increased between the PVA and the surrounding cartilage, but reduced on the PVA surface. In the control group, reduced staining was observed throughout. Scanning electron microscopy showed increased bone tissue in the lower n-HA+PA66 layer that was in close approximation with the upper PVA layer of the composite. In the PVA group, the bone tissue around the material had receded, and in the control group, the defect was filled with bone tissue, while the superior aspect of the defect was filled with disordered, fibrous tissue.

**Conclusion:**

The diphase biological composite material PVA/n-HA+PA66 exhibits good histocompatibility and offers a satisfactory substitute for articular cartilage and subchondral bone.

## Background

Articular cartilage is a type of hyaline cartilage characterized by low friction, good elasticity, and hypertonicity that has an important role in maintaining joint movement. Trauma and osteoarthritis are the two factors that can lead to articular surface defects, which eventually develop into joint dysfunction. Joint fusion or joint replacement is necessary to repair joint dysfunction, because articular cartilage itself lacks a regenerative capacity [1, 2]. Adult articular cartilage has a limited reparatory capability; that is, it can partially or totally repair itself when the defect diameter is less than 3 mm but not when the diameter exceeds 3 mm, in which case it will develop into arthritis [3]. Joint replacement is not ideal for young and middle-aged patients because the joint implant has a limited life expectancy. For this reason, an effective method for repairing articular cartilage defects is an emergent issue in the field of orthopedics.

Previous methods used to repair articular cartilage defects include enhancement of the articular cartilage healing capacity and biological repair by autologous or xenogenic tissue grafting. The latter method includes autologous periosteal, perichondrial, and osteochondral grafting as well as allogeneic osteochondral grafting. But the long-term therapeutic effects of these methods are not satisfactory [4]. With advances in tissue engineering technology, some progress has been made in the repair of articular cartilage defects. Nevertheless, repair of articular cartilage to a high standard, with satisfactory long-term therapeutic effects, has not been acquired in the clinic [5, 6]. At present, the artificial materials used in the treatment of advanced osteoarthrosis are made of metal, ceramics, and ultra-high molecular weight polyethylene, with increasing attention being paid to the postoperative complications caused by implant abrasion and loosening. Therefore, some researchers are investigating alternative artificial cartilage substitutes as a repair strategy [7].

Elastomeric materials with a similar biomechanical property to cartilage have been mostly selected as alternative materials for cartilage replacement therapies, such as silicon rubber, polyurethane, and polyvinyl alcohol (PVA) hydrogel. Silicon rubber is no longer a popular material choice, owing to its ease of abrasion and absorption of oil in the body [8]. Polyurethane, as a long-term implant material, requires an improvement in its degradation rate. Moreover, the hydrolysate of the curing agent diisocyanate is a potential carcinogenic substance [9]. Polyvinyl alcohol (PVA) hydrogel, on the other hand, is a porous material similar to natural cartilage and is considered by some to be an ideal alternative material to cartilage [10]. It is a water-soluble synthetic polymer hydrolyzed by polyvinyl acetate that has been widely used as artificial cartilage, drug delivery systems, artificial meniscus, heart valve, anti-thrombin materials, vascular grafts, and biomedical sponges in the field of biomedicine for its high elasticity, stable chemical property, ease of molding, absence of toxicity and adverse reactions, and good histocompatibility within the human body [11–16].

Despite its suitable properties, the use of the PVA hydrogel in an articular cartilage and subchondral bone defect remains a concern for orthopedists. Previously, the implanted PVA was fixed by sticking or sutures, which produced poor fixation and led to the formation of a gap between the PVA and the subchondral bone. Fabricating the PVA biomaterial into a cylinder, which can be inserted into the articular cartilage and subchondral bone, offers poor stability to the joint. Likewise, mechanical fixation and chemical fixation have also yielded unsatisfactory outcomes [17, 18]. Therefore, the application of PVA for the repair of articular cartilage defects is limited.

In this study, we prepared a novel artificial bone material comprising nano-hydroxyapatite (n-HA) and polyamide 66 (PA66) (n-HA+PA66) to form a base for insertion of the PVA hydrogel. This double-layered biomaterial PVA/n-HA+PA66 composite was prepared using the in situ synthesis and freeze-thaw cycle method. The upper layer of the biomaterial composite was made of PVA and used as a substitute for articular cartilage, while the lower layer was made of n-HA+PA66 and used to substitute for subchondral bone. The upper layer and lower layer were firmly connected prior to implantation in a rabbit patellofemoral defect model to assess its potential use as a therapeutic grafting substitute.

## Methods

### Materials

Biomaterial composites PVA/n-HA+PA66 and PVA were provided by the Nanometer Analytical and Testing Center, Sichuan University, China.

### Animals

This study was performed at the Laboratory of Tissue Engineering, Department of Orthopedics, Huaxi Hospital, Sichuan University, China between July 2014 and July 2016. Seventy-two healthy adult New Zealand rabbits, aged 5–8 months, weighing 160–240 g, and with an equal number of males and females, were provided by the Laboratory Animal Center, Sichuan University, China (license No. SCXK (Chuan) 2004–14).

### Model preparation and group management

Seventy-two rabbits (36 male and 36 female) were randomly divided into three groups with 24 rabbits in each group: PVA/n-HA+PA66 implant (contains 11 male and 13 female), PVA implant (contains 14 male and 10 female), and control (contains 11 male and 13 female). Following anesthesia by an intraperitoneal injection of 30 g/L pentobarbital, an incision was made on the skin around the medial patella to expose the patellofemoral joint. The femoral bone joint was perforated (diameter 5 mm, depth 5 mm) to prepare models of articular cartilage and subchondral bone defects. The double-layer biomaterial composite PVA/n-HA+PA66 and simple PVA were implanted into the hole, respectively, and lightly pressed through the use of an inserter to stabilize the implant, the control group received no treatment. Each rabbit was intramuscularly injected with 40,000 units of penicillin per day for 3 days after surgery. After wound sutures, the operated limbs were not immobilized and allowed to move freely.

### Gross observation

After surgery, the diet, activity level, wound healing, and range of motion were assessed for each rabbit. At a postoperative time period of 4, 8, 12, or 24 weeks, rabbits were sacrificed under anesthesia and the articular cavity was opened to examine the cartilage surrounding the implant, synovial membrane, and implant stability and compared with the contralateral joint surface.

### Histomorphological observation

Eighteen rabbits were sacrificed at postoperative 4 weeks for histomorphological observation, 18 rabbits were sacrificed at postoperative 8 weeks, and 9 rabbits were sacrificed at 12 and 24 weeks postoperatively for histomorphological observation and immunohistochemical detection. Nine rabbits were sacrificed at 12 and 24 weeks postoperatively for scanning electron microscopy observation. Following removal of condyles of the femur, a tissue specimen about 1 cm in length was harvested, with the implant as a center, and fixed with 40 g/L paraformaldehyde. Specimens were then decalcified with 10% EDTA and dehydrated and embedded with paraffin following routine procedures. Slices of 5-μm-thick sections were stained with hematoxylin-eosin, Masson’s trichrome, and toluidine blue and observed under the microscope (BX51, Olympus, Tokyo, Japan).

### Immunohistochemical detection of type II collagen

At postoperative 8, 12, and 24 weeks, the tissue specimens were harvested as described above and then fixed, decalcified, hydrated, embedded, sliced into 5-μm-thick sections, and stained for type II collagen expression using standard immunohistochemistry. The specimens were then assessed using an inverted phase contrast microscope (BX51; Olympus).

### Scanning electron microscopy observation

At postoperative 12 and 24 weeks, tissue specimens (1 × 1 × 1 cm) were fixed with 2.5% glutaraldehyde (pH 7.2–7.4), dehydrated with ethanol series, and subject to gold sputtering. The interface between the implant and the surrounding cartilage and subchondral bone was observed by scanning electron microscopy (S3400N; Hitachi, Japan).

## Results

### Appearance and inner structure of biomaterial composite

**Fig. 1:**
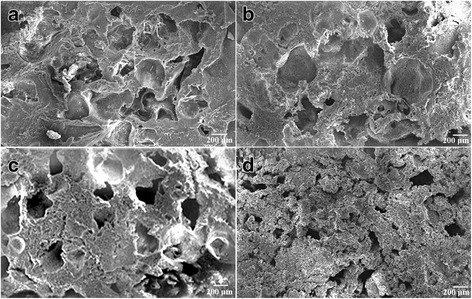
Scanning electron microscopy image of the porous biomaterial nano-hydroxyapatite+polyamide 66 (×60) (**a** x80, **b** x70, **c** x60, **d** x50)

The cylindrical PVA/n-HA+PA66 composite was 5 mm in diameter and 5 mm in height. The upper layer of PVA was 1.5 mm high and consisted with an ivory, smooth, and tenacious surface. By comparison, the lower layer of n-HA+PA66 was 3.5 mm high, and had a rough and stiff composition. Using scanning electron microscopy, the PVA exhibited a porous structure, with a pore diameter of 5–40 μm, a porosity of 75.3% and water ratio of 71.6%. The n-HA+PA66 component of the biomaterial was also porous, with a pore diameter of 100–400 μm and porosity of 61.7%. By X-ray diffraction analysis, the PVA appeared as a semi-crystalline polymer, with strong hydrogen bonds between the hydroxyl groups. The composite n-HA+PA66 consisted of a uniform crystal structure, with firm bonds to polyamide (Fig. 1).

### Biological behaviors of rabbits

Seventy-two New Zealand rabbits were included in the final analysis. After surgery, no rabbits had died or suffered wound infection/secretion. The rabbits in the PVA/n-HA+PA66 implant group and PVA implant group showed a good range of motion. In the control group, joint motion was reduced in two rabbits and this symptom developed into knee joint stiffness at 8 weeks after surgery.

### Changes in the knee joint cavity

**Fig. 2:**
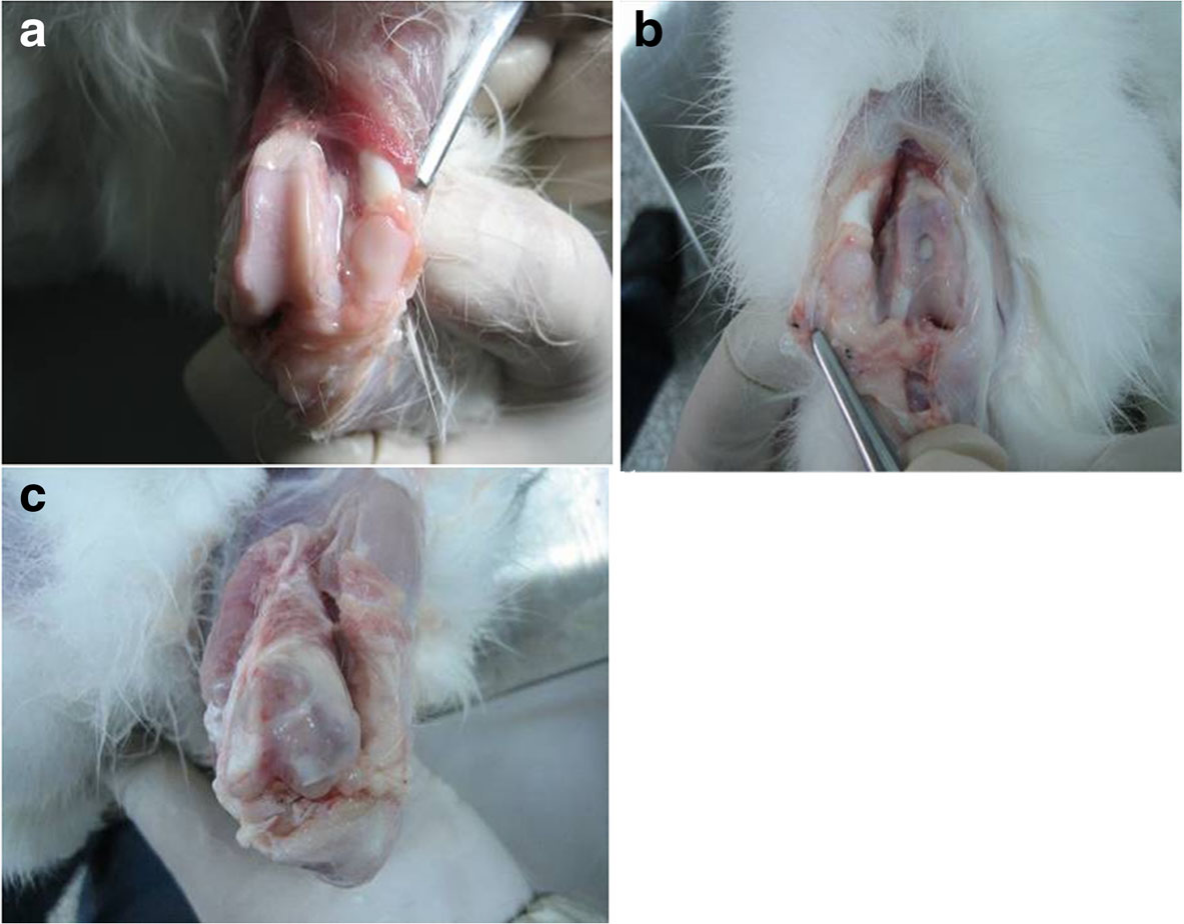
At 24 weeks after implantation of polyvinyl alcohol/nano-hydroxyapatite and polyamide 66. No obvious changes were observed in the peripheral articular cartilage; the contralateral articular surface was smooth (**a**). At 24 weeks after implantation of polyvinyl alcohol, the implant had dropped and was not firmly fastened into the joint (**b**). At 24 weeks in the control group, the surface of joint appears rough and articular cartilage in the surrounding region presented with degenerative changes (**c**)

In the PVA/n-HA+PA66 implant group, the articular surface was smooth. At 4 weeks, the gap surrounding the implant was filled with fibrous tissue, which secured the implant such that it could not be pulled out. At 8, 12, and 24 weeks, the gap surrounding the implant disappeared, and the joints regained good function. No obvious cartilage degeneration was observed, and the implant bonded well with the adjacent articular cartilage and subchondral bone. As expected, the contralateral articular surface was smooth without abrasion. No hyperplastic and plump synovial membrane formations were observed (Fig. 2a). In the PVA implant group, the surface of the implant was smooth at 4 weeks, but the implant was not firmly fastened and easily prolapsed. At 8 and 12 weeks, the implant had dropped by 2.0–3.0 mm. At 24 weeks, a small amount of degenerative change was observed in the cartilage (pale color and surface layer abrasion), and the implant was not firmly fastened into the joint (Fig. 2b). In the control group, the defect region was filled with fibrous granulation tissue at 4 weeks, which gradually enclosed the articular surface. Light colored, fibrous cartilage with a roughened surface was identified. At 24 weeks, the fibrous tissue had calcified and continued to appear rough. The articular cartilage in the surrounding region presented with degenerative changes, and the articular synovium exhibited hyperplasia, swelling, and appeared plump (Fig. 2c). Severe traumatic arthritis, articular synarthrophysis, and stiffness occurred in two of the rabbits in this group.

### Histomorphological observations

**Fig. 3:**
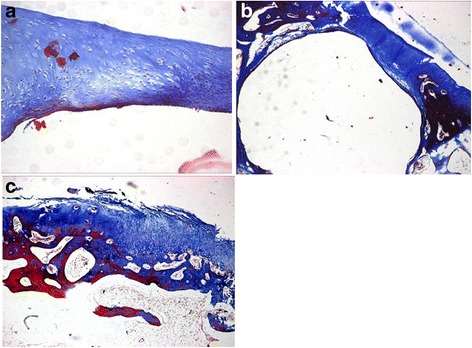
At 24 weeks after implantation of polyvinyl alcohol/nano-hydroxyapatite and polyamide 66, part of the polyvinyl alcohol surface was covered by collagen (×40) (**a**). At 24 weeks in the PVA implant group, the adjacent cartilage had thinned and continued to show degenerative changes (×40) (**b**). At 24 weeks in the control group, the fibrous tissue was poorly arranged and had formed transparent cartilage (×40) (**c**)

At 4 weeks, for rabbits in the PVA/n-HA+PA66 implant group, the fibrous tissue in the gap between the implant and adjacent tissue contained a small number of filtrated inflammatory cells, with some fibrous tissue growing into the pores of the n-HA+PA66 biomaterial. At 8 and 12 weeks, a large amount of fibrous tissues had infiltrated into the pores of the n-HA+PA66 lower layer, with the formation of some collagen and osteoid. The gap between the PVA in the upper layer and the adjacent cartilage had become filled with articular cartilage that had also started to cover the PVA surface. At 24 weeks, the lower layer had bonded with the adjacent tissue, with much osteoid in the pores that had formed a reticular layer. No abrasion to the PVA in the upper layer was observed, and the adjacent cartilage was ordered, covering the surface of the PVA, with no obvious degeneration (Fig. 3a).

In the PVA implant group, there was a large number of infiltrated inflammatory cells at 4 weeks in the gap between the implant and the adjacent subchondral bone, with some proliferative fibrous tissue. At 8 and 12 weeks, the gap between the implant and the host tissue was filled with fibrous tissue. The biomaterial adjacent to the articular cartilage edge appeared blunted and presented with slight degenerative changes. At 24 weeks, the adjacent cartilage had thinned and continued to show degenerative changes (Fig. 3b). The fibrous tissue in the defect site of rabbits in the control group was interspersed with a large number of fibroblasts and inflammatory cells at 4 weeks. At 8 and 12 weeks, the granulation tissue had become calcified, and by 24 weeks, the majority of the defect region had formed bony trabeculae and osteoid. In the upper layer, the fibrous tissue was poorly arranged and had formed transparent cartilage; the adjacent host cartilage had thinned and begun to degenerate (Fig. 3c).

### Immunohistochemical detection of type II collagen

**Fig. 4:**
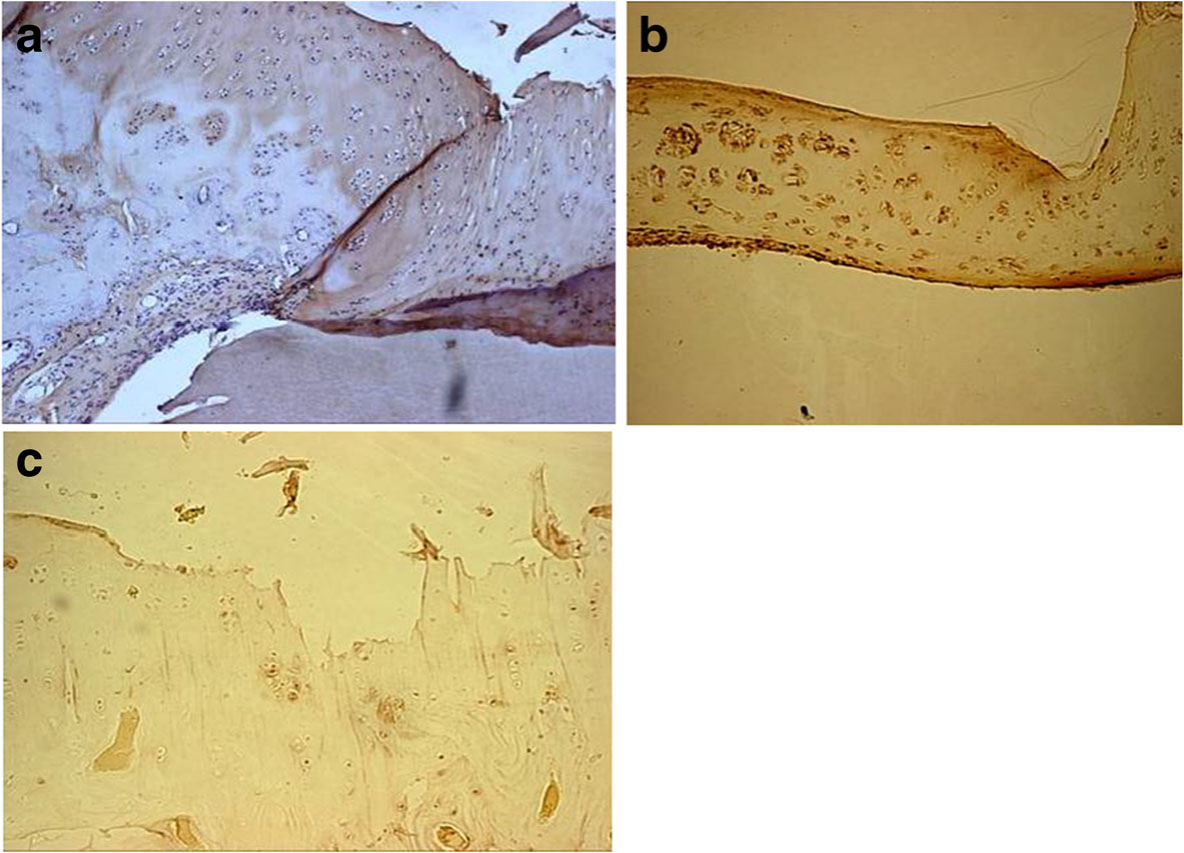
At 24 weeks in PVA/n-HA+PA66 implant group, immunohistochemical detection of type II collagen showed positive staining on the surface and in the peripheral region of the polyvinyl alcohol implant (×100) (**a**). At 24 weeks in the PVA implant group, weak type II collagen staining was observed in the gap between the PVA biomaterial and the adjacent tissue(×100) (**b**). At 24 weeks in the control group, a small amount of type II collagen was present, presenting as weak positive staining on the surface of the defect (×100) (**c**)

Positive type II collagen staining was found in the PVA/n-HA+PA66 implant group at 12 weeks in the gaps between the PVA and adjacent articular cartilage and on the surface of PVA biomaterial. At 24 weeks, type II collagen staining had increased at the cartilaginous margin and on the surface of the PVA biomaterial (Fig. 4a). For rabbits in the PVA implant group, weak type II collagen staining was observed in the gap between the PVA biomaterial and the adjacent tissue, with positive staining on the surface of the PVA and in the adjacent cartilage. There was a slight increase in this staining at 24 weeks, but the staining on the surface of PVA biomaterial and in the adjacent cartilage was attenuated (Fig. 4b). In the control group, a small amount of type II collagen was present at 12 weeks, presenting as weak positive staining on the surface of the defect and in the adjacent articular cartilage. At 24 weeks, type II collagen staining was attenuated on the surface of the defect and in the adjacent articular cartilage (Fig. 4c).

### Scanning electron microscopy observation

**Fig. 5:**
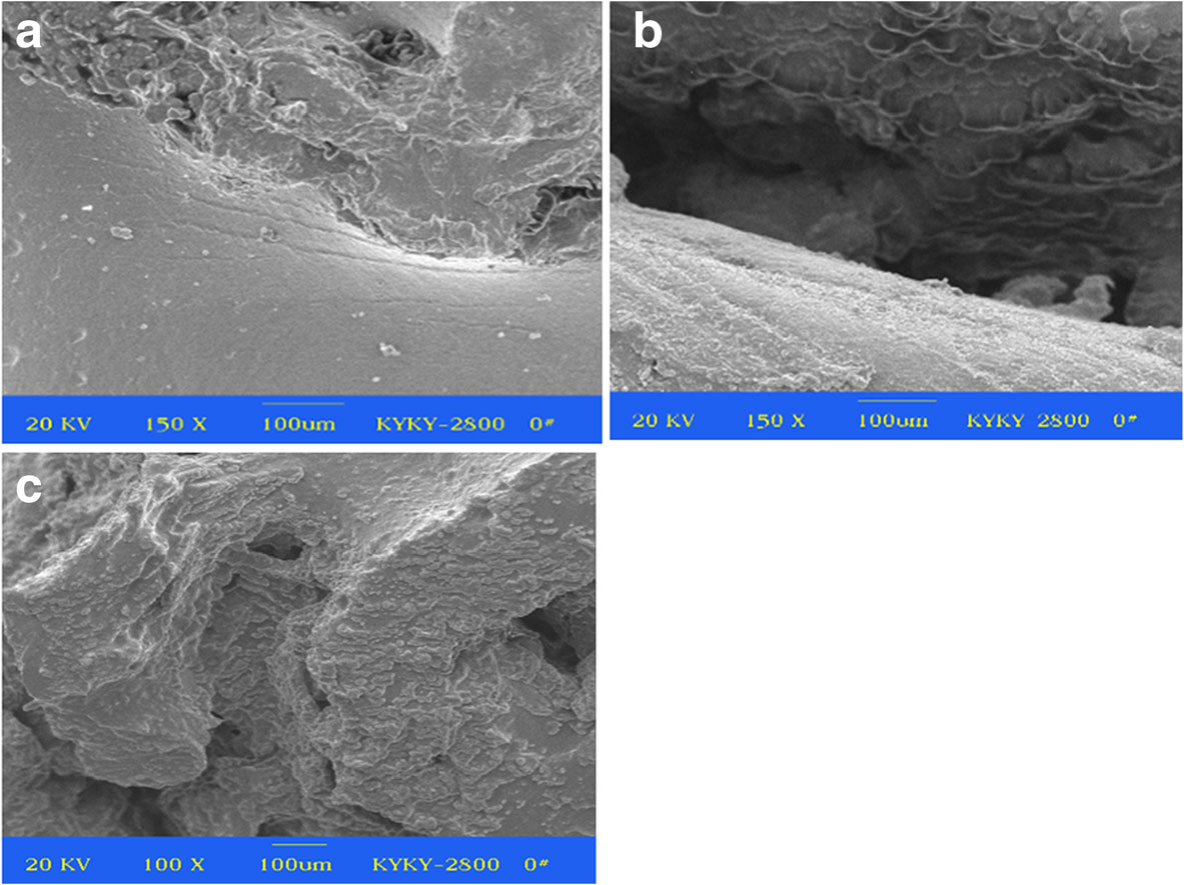
At 24 weeks postoperatively, the n-HA+PA66 biomaterial in the lower layer was tightly connected with adjacent tissue and the PVA biomaterial in the *upper* layer was tightly connected with the n-HA+PA66 biomaterial in the *lower* layer (SEM × 150) (**a**). In the PVA implant group, a poor connection and gap with the adjacent tissue were observed (SEM × 150) (**b**). In the control group, the *lower* part of defect was filled with sclerotin and the *upper* part contained a disordered, scar-like fibrous tissue (SEM × 150) (**c**)

In the PVA/n-HA+PA66 implant group, a large number of fibroblasts were found in the pores of n-HA+PA66 biomaterial in the lower layer at 12 weeks, along with substantial osteoid. The osteoid was arranged in strips, and the gap between the n-HA+PA66 biomaterial and adjacent tissues contained numerous fibroblasts and collagen, with some calcified fibrous tissue surrounding the PVA in the upper layer. At 24 weeks, the n-HA+PA66 biomaterial pores in the lower layer contained osteoid that made connections with the adjacent tissue, and the PVA in the upper layer was tightly connected with the n-HA+PA66 biomaterial in the lower layer (Fig. 5a). By comparison, in the PVA implant group, a poor connection with the adjacent tissue was observed at 12 weeks, with an obvious margin. Part of the PVA in the upper layer was covered by fibrous strips. At 24 weeks, part of the adjacent sclerotin had pulled away, enlarging the gap (Fig. 5b). In the control group, the lower part of defect region was filled with solid sclerotin and the surface was covered by a layer of fibrous tissue at 12 weeks. At 24 weeks, the lower part of the defect was filled with sclerotin, and the upper part contained a disordered, scar-like fibrous tissue (Fig. 5c).

## Discussion

Articular cartilage is a tissue without blood vessels, lymphatic vessels, and nerves. As such, chondrocytes under general circumstances have limited capacity to undergo mitosis or regenerate the cartilage in response to damage or deterioration. According to the structure and characteristics of cartilage, a substitute material used as artificial cartilage should meet the following requirements: good biomechanical property, excellent lubricity and wear resistance, ability to induce chondrocyte growth, firm connection with bone base, and biocompatibility.

### Histocompatibility of composite biomaterial PVA/n-HA+PA66

PVA hydrogel exhibits physical properties that are more similar to in vivo tissue than many other artificial composites. First, its expansive capability and water permeability contribute to its overall satisfactory biocompatibility [19]. Second, its flexibility and elasticity can reduce the load experienced by surrounding cells and tissue. Third, PVA exhibits a good biomechanical property, which is similar to the elastic modulus of cartilage, and has a small surface friction coefficient [20]. Therefore, PVA hydrogel is currently considered a good substitute biomaterial for articular cartilage [21–23].

Some scholars have performed studies to assess the cellular toxicity, safety, and excretion of PVA. Strong evidence exists that PVA does not cause hemolysis, allergic response, or skin irritations [24]. Our results demonstrate that PVA exhibited good compatibility with the adjacent articular cartilage; after the PVA implantation, the adjacent articular cartilage did not present with any degenerative changes, type II collagen was secreted, and chondrocytes were arranged in order. After 4 weeks, some chondrocytes were observed on the PVA surface and filled the gap between the PVA and the adjacent articular cartilage. At 24 weeks, the articular cartilage surrounding the PVA grew well and did not present with any obvious degenerative changes, showing positive type II collagen staining on the surface and at the biomaterial edge, suggestive of cartilage growth. These findings indicate that PVA exhibits good biocompatibility with adjacent host articular cartilage.

HA has a good osteoconductivity and has been well accepted as a bone repair substitute [25]. PA66 is a polymer with strong intensity, high flexibility, and good stability. Previous studies have shown that the combination of these two materials yields a high molecular weight polymer, n-HA+PA66, that was initially prepared under international advanced standards using Chinese intellectual property. In this study, according to human bone tissue compositions, we found that our novel biomaterial exhibited the strong rigidity of n-HA and the highly flexible nature of PA66, thereby generating a structure with similar properties to the bone and articular cartilage that was appropriate for presenting the PVA biomaterial [26–28]. Zhang et al. [29] also evaluated the biological characteristics of the n-HA/PA66 composite biomaterial in vivo and in vitro, showing that the n-HA/PA66 composite biomaterial did not dissolve in the blood and induced no cell toxicity, skin irritation or allergic response, and no pyrogen reaction or other adverse reactions after intramuscular implantation for 90 days or bony implantation for 180 days. Using this composite to repair dog mandibular cortical defects, Zheng et al. [30] found that, after surgery, the wound healed well, there were no rejections, the implant made strong connections with the bone tissue, and displayed good osteoconductivity, suggesting good biocompatibility and biological activity of the material.

### Integrated composite material formation by firm connection of upper and lower layers of interfaces

The PVA/n-HA+PA66 composite consisted of a uniform crystal structure, with firm bonds to polyamide, as evidenced by electron microscopy (Fig. 1). PVA and n-HA+PA66 can be integrated by freeze-thaw cycles and casting because of the porosity of the n-HA+PA66 compound, which is suitable for permeation of liquid compositions. Part of the dissolved PVA compositions casted onto the n-HA+PA66 can directly permeate into the pores of n-HA+PA66. PVA and n-HA+PA66 form a steady connection after repeated freeze-thaw cycles.

### Stability and advantages of integrated composite material after implantation

Under normal conditions, subchondral and cancellous bones below the articular cartilage play an important role in protecting the articular cartilage from high stress. When the joints are exposed to high loads, the subchondral bone assigns the majority of this stress to the cancellous bone, which is arranged in a radial manner to greatly decrease the stress to articular cartilage. Brittberg et al. [31] reported that cartilage defects often involve the subchondral bone; they result in destruction of the normal cartilage and induce subsequent changes to the mechanical properties of the joint, leading to a decreased healing rate and degenerative changes. For this reason, the PVA hydrogel used as a substitute of articular cartilage should provide sufficient support to the subchondral and cancellous bones. However, a good connection between the PVA hydrogel and the bone has, until now, remained problematic. Recently, Gu et al. [32] used mechanical-chemical methods to investigate a firm connection between PVA hydrogel and metal (as a bottom layer for bone). In addition, Oka et al. [33] implanted PVA-H into the cartilage defect region and fixed it using tissue grown in titanium mesh pores. Some therapeutic effects were acquired, but the long-term biomechanical properties of these composites deserve further investigation.

In this study, we designed a novel artificial cartilage substitute biomaterial to solve this problem with PVA presentation for joint repair. The upper layer was made of PVA and the lower layer of n-HA+PA66, integrated by freeze-thaw cycles and casting. The upper layer functioned to substitute for articular cartilage while the lower layer substituted as a subchondral bone. The lower layer exhibited a porous structure to facilitate the ingrowth of fibrous and bony tissue. The formation of biological self-locking fixation of the n-HA+PA66 after implantation can effectively prevent displacement of the implant and greatly improve the stability and function of the artificial cartilage upper layer of the biomaterial. PVA exhibits a good stability throughout the entire lifespan, which plays a positive role in its functioning as articular cartilage.

Our results confirmed that the composite PVA/n-HA+PA66 immediately fixed into the defect, becoming integrated with the adjacent subchondral bone. At 4 weeks after implantation, the lower part of the biomaterial showed preliminary connections with the adjacent tissue. After 8 weeks, n-HA+PA66 in the lower layer attained good connections with the adjacent tissue, that gradually calcified into bone tissue. Thus, the n-HA+PA66 in the lower layer offers a strong support to the upper PVA and contributes to PVA functioning as a substitute articular cartilage.

Our results showed that the PVA alone lacked the necessary support to subchondral bone, being extremely unstable and easily deformable under the influences of external forces owing to lacking of good fixation. Electron microscopy results showed an obvious boundary between the entire PVA and the adjacent tissue that likely arose because the bone tissue and fibrous tissue cannot grow into the inner portion of the PVA. This leads to poor interconnectivity, displacement of PVA biomaterial, increased PVA abrasion, a non-uniform stress distribution, and finally degeneration of adjacent cartilage. It may even lead to osteophyma.

## Conclusion

Taken together, the composite biomaterial PVA/n-HA+PA66 serves as a good integrated biomaterial substitute for articular cartilage and subchondral bone. The implanted PVA/n-HA+PA66 can reconstruct the smooth cartilage surface, reduce abrasion, postpone or prevent the occurrence of osteoarthritis, and provide an alternative biomaterial for repair of articular cartilage defects. However, the problem of abrasion in long-term application of PVA biomaterial deserves further investigation.

## Abbreviations

n-HA+PA66: nano-hydroxyapatite (n-HA) and polyamide 66 (PA66)
PVA: Polyvinyl alcohol hydrogel
